# First evidence of hybridization between golden jackal (*Canis aureus*) and domestic dog (*Canis familiaris*) as revealed by genetic markers

**DOI:** 10.1098/rsos.150450

**Published:** 2015-12-02

**Authors:** Ana Galov, Elena Fabbri, Romolo Caniglia, Haidi Arbanasić, Silvana Lapalombella, Tihomir Florijančić, Ivica Bošković, Marco Galaverni, Ettore Randi

**Affiliations:** 1 Department of Biology, Faculty of Science, University of Zagreb, Rooseveltov trg 6, Zagreb 10000, Croatia; 2 Laboratorio di Genetica, Istituto Superiore per la Protezione e la Ricerca Ambientale (ISPRA), Ozzano dell’Emilia (BO) 40064, Italy; 3 Department of Biological, Geological and Environmental Sciences University of Bologna, Via Selmi 3, Bologna 40126, Italy; 4 Department for Hunting, Fishery and Beekeeping, Faculty of Agriculture in Osijek, Josip Juraj Strossmayer University of Osijek, Kralja Petra Svačića 1d, Osijek 31000, Croatia; 5 Department 18/Section of Environmental Engineering, Aalborg University, Sohngårdsholmsvej 57, Aalborg 9000, Denmark

**Keywords:** *Canis*, interspecific hybridization, gene introgression, major histocompatibility complex, melanism, β-defensin CDB103

## Abstract

Interspecific hybridization is relatively frequent in nature and numerous cases of hybridization between wild canids and domestic dogs have been recorded. However, hybrids between golden jackals (*Canis aureus*) and other canids have not been described before. In this study, we combined the use of biparental (15 autosomal microsatellites and three major histocompatibility complex (MHC) loci) and uniparental (mtDNA control region and a Y-linked *Zfy* intron) genetic markers to assess the admixed origin of three wild-living canids showing anomalous phenotypic traits. Results indicated that these canids were hybrids between golden jackals and domestic dogs. One of them was a backcross to jackal and another one was a backcross to dog, confirming that golden jackal–domestic dog hybrids are fertile. The uniparental markers showed that the direction of hybridization, namely females of the wild species hybridizing with male domestic dogs, was common to most cases of canid hybridization. A melanistic 3bp-deletion at the *K* locus (*β*-defensin CDB103 gene), that was absent in reference golden jackal samples, but was found in a backcross to jackal with anomalous black coat, suggested its introgression from dogs via hybridization. Moreover, we demonstrated that MHC sequences, although rarely used as markers of hybridization, can be also suitable for the identification of hybrids, as long as haplotypes are exclusive for the parental species.

## 1. Introduction

Interspecific hybridization is relatively frequent in nature, occurring not only in plants but also in animals, where at least 10% of species are involved in admixture and potential introgression [1]. Interspecific hybridization in *Canis* has been described in a number of studies in North America (e.g. coyote–grey wolf [2]). Moreover, cases of anthropogenic hybridization between wild canids and widespread free-ranging domestic dogs are particularly alarming, because they may threaten the survival of endangered species (e.g. the Ethiopian wolf, *Canis simensis* [3]), or may deeply change the genetic make-up of wild populations in human-dominated landscapes (e.g. grey wolf [4–6]).

The golden jackal (*Canis aureus*) is a medium-sized species, currently distributed in northern and northeastern Africa, southeastern Europe and large parts of southern Asia [7]. European golden jackals were first reported in 1491 in the coastal region of southern Dalmatia, where they still occur. After suffering a severe decline in the first half of the twentieth century, the European population has recovered and has been expanding since the early 1980s, especially in the Balkan regions [8].

To our knowledge, there are no published cases of recent hybridization between golden jackals and any other canid species in nature, although genome-wide traces of ancestral admixture with wolves have been recently documented [9], and the assignment of the African *Canis aureus lupaster* to the wolf clade cannot rule out the hypothesis of ancient hybridization events [10,11]. The only two documented cases of present-day hybrids were questionable, since they were only based on morphological measurements: in Romania a putative jackal–wolf hybrid was shot in 1942; in Hungary, the skull of a suspected jackal–dog was discovered in 1983, but erroneously reported as the first jackal shot in the country after 41 years [12].

Molecular techniques are routinely used for the identification of closely related species and their hybrids within the first two to three generations of admixture [6,13]. The combined use of maternal and paternal markers may also reveal the hybridization direction [6,14–16]. The mitochondrial DNA control region (mtDNA CR) can identify the maternal ancestry in putative hybrids [15,17]. A single mtDNA CR haplotype has been found so far in European golden jackal populations [18,19], facilitating the identification of golden jackal–dog hybrids through the maternal line. Paternal ancestry of hybrids may be revealed by species-specific Y chromosome markers developed to discriminate between golden jackal and domestic dog males, based on an insertion found in a dog *Zfy* intron, but not in golden jackal [20]. In addition, putatively neutral biparental markers such as microsatellite loci (STR), originally developed for the domestic dog, can efficiently cross-amplify and identify golden jackal genotypes [19,21], thus providing estimates of the proportion of neutral admixture inherited from the mixing parental populations (e.g. [5,17,22]).

Functional markers could further contribute to the investigation of hybridization and introgression. The major histocompatibility complex (MHC) is a multigene family that is commonly used in adaptive variation studies [23]. MHC genes encode cell-surface glycoproteins that bind and present antigens to T cells, which trigger an appropriate immune response, thus playing a pivotal role in the vertebrate immune system. However, to our knowledge, MHC genes have been used as markers to detect hybridization in only two vertebrate species: a case of hybridization between Iberian ibex and domestic goat [24], and a study of introgression of dog MHC alleles in wild-living Italian wolves [25]. Other functional mutations that could be used in determining phenotypical variation have been discovered in recent genomic studies [26], such as a dominant three-nucleotide deletion in the *β*-defensin CBD103 gene (the *K* locus) correlated to black coat colour in canids, which could have been introduced from dogs into wild-living wolves in North America [27] and in Italy [16] via hybridization (hereafter referred to as ‘melanistic deletion’).

Thus, the primary objective of this study was to document the potential occurrence and directionality of golden jackal–dog hybridization in an expanding jackal population, using combined genetic analyses of 15 microsatellite loci, mtDNA control region and a Y chromosome marker. Our secondary objectives were to assess the presence of a functional melanistic deletion at the *β*-defensin CBD103 gene in an individual with black coat coloration and to test the applicability of coding markers such as MHC genes to detect hybridization between closely related species, such as the dog and the golden jackal.

## 2. Material and methods

### 2.1 Putative golden jackal–dog hybrid samples

**Figure 1.**
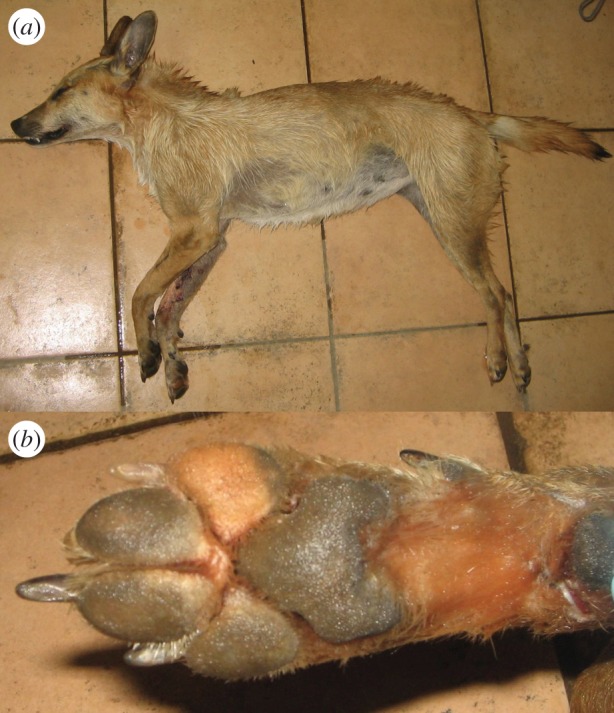
Female golden jackal–dog hybrid (S21) (*a*) and its forelimb with notable digital pad depigmentation (dog characteristic) and partially joined digital pads of the middle fingers (golden jackal characteristic) (*b*).

**Figure 2.**
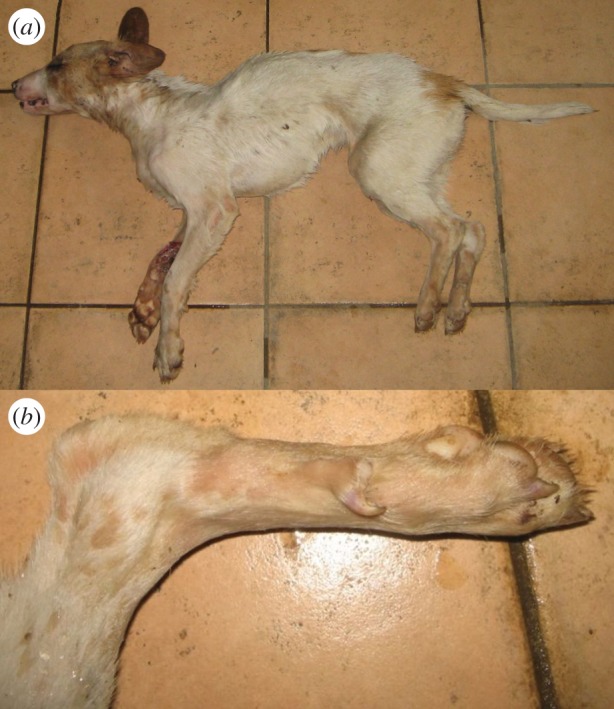
Male golden jackal–dog hybrid (S22) (*a*) and its hind leg with dewclaw (*b*).

**Figure 3.**
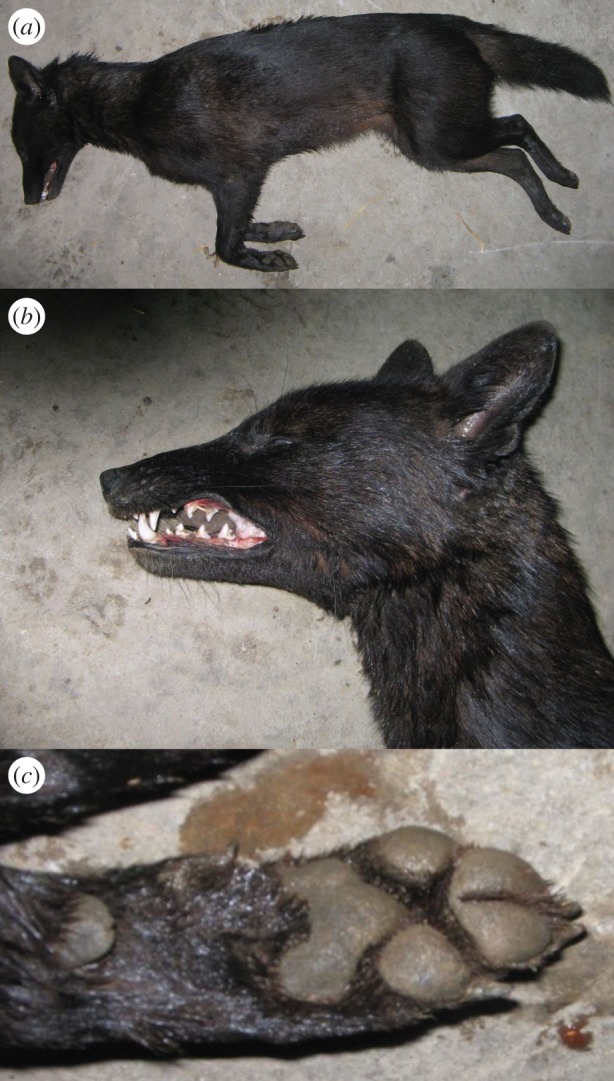
Male golden jackal–dog hybrid (60c) with black coat coloration (*a*) and ears with rounded tip (*b*) (dog characteristics), and forelimbs with partially joined digital pads of the middle fingers (golden jackal characteristics) (*c*).

We collected muscle tissue samples from three putative golden jackal–dog hybrids legally harvested by hunters in Croatia. The putative hybrids were initially identified on the basis of their unusual morphological traits. The individual S21 (figure 1) was an adult female showing light coat colour, digital pad depigmentation and atypical long ears with rounded tip (whereas golden jackals have shorter triangle-shaped ears). However, the digital pads of the middle fingers were partially joined as commonly occurs in golden jackals, but not in dogs [12] (I. Bošković 2012, unpublished data). The individual S22 (figure 2) was a juvenile male found and shot together with female S21, probably representing a mother–son pair. It displayed a dog-like morphology, particularly similar to the Istrian shorthaired hound breed (very short hair on the head, white coat colour with sparse patches of light brown, dewclaws on hind legs). However, the animal’s tail was shorter and thicker than typical for this dog breed and more similar to that of a golden jackal. Thus, we hypothesized it could have originated through a backcross between the putative hybrid female S21 and a male Istrian shorthaired hound dog. The third individual (60c) (figure 3) was a male that exhibited black coat coloration, atypical for golden jackals, and other dog characteristics such as ears with rounded tip. However, the digital pads on the middle fingers of its forelimbs were partially joined, as in golden jackals.

Tissue samples were stored in 96% ethanol at −20°C prior to analyses. DNA was extracted using a Wizard Genomic DNA Purification kit (Promega, USA).

### 2.2 Reference samples

To correctly identify the origin and ancestry of the putative golden jackal–dog hybrids, we used as reference source populations 50 jackal samples from Croatia [19] and 51 mixed breed dog samples from Croatia, previously genotyped at STR markers. Reference jackal samples were legally shot or road-killed in Croatia.

### 2.3 Mitochondrial DNA analysis

We amplified the hypervariable left domain of the mtDNA CR using primers L-Pro [28] and H-576 [29] (electronic supplementary material, table S1). The polymerase chain reactions (PCRs) were carried out in 30 μl containing 1× Qiagen HotStarTaq Master Mix (Qiagen HotStarTaq Master Mix Kit, Qiagen, USA), 0.2 μM of forward and reverse primer and 3 μl template DNA. Cycling conditions were the following: 95°C for 15 min, 35 cycles of 40 s at 94°C, 50 s at 55°C, 1 min at 72°C, and 10 min final extension at 72°C. The amplification products were purified (Wizard SV Gel and PCR Clean-Up System, Promega) and sequenced using L-Pro primer. Sequences were aligned in BioEdit [30] with the only golden jackal haplotype found so far [18,19] and 12 dog haplotypes found among mixed breed dogs from Croatia [31].

### 2.4 Y chromosome analysis

We analysed the two male samples S22 and 60c using a PCR-based Y chromosome marker method in which the dog DNA template produces two amplicons, whereas the golden jackal template produces only one [20] (electronic supplementary material, table S1).

### 2.5 Autosomal microsatellite loci analysis

We genotyped the reference populations and the putative hybrid samples at 15 unlinked autosomal canine STRs using the same procedure described in Fabbri *et al*. [19] (electronic supplementary material, table S2). The average number of alleles (*N*_a_) and private alleles (*N*_p_), and the observed and expected heterozygosity (*H*_o_, *H*_e_) were estimated using GenAlEx v. 6.5 [32,33] as measures of genetic diversity. Exact tests for Hardy–Weinberg equilibrium were computed using the Guo and Thompsons’ Markov chain method [34] as implemented in the software Genpop v. 4.00 [35]. The sequential Bonferroni correction test for multiple comparisons was used to adjust significance levels [36]. The 15-STR multilocus genotypes of the reference jackals and dogs, and of the three putative hybrids were used to distinguish species and to detect putative admixed individuals and their ancestry through two different methodologies: (i) a multivariate analysis: principal coordinates analysis (PCoA) of individual STR genotypes implemented in GenAlEx [37]; (ii) a Bayesian clustering procedure implemented in Structure v. 2.3.4 [38–40], which estimates the admixture proportion of each individual genotype.

We used the Admixture model with independent allele frequencies (*I*-model) running five replicates of *K* from 1 to 5 using 5×10^5^ iterations of MCMC following a burn-in period of 5×10^4^ iterations. The optimal number of populations *K* was determined according to Evanno *et al*. [41] independently from any prior non-genetic information (option usepopinfo not active). For each group, we assessed the average proportion of membership (*Q*_i_) to each different clusters, and individual assignment was consequently based on the proportions of membership (*q*_i_) estimated for every single individual. Based on these first Structure results, admixture analyses were performed again assuming two reference groups (jackal and dog) for the assignment of the putative jackal–dog hybrids (PHy). Structure was run with *K*=2, with the option ‘usepopinfo’ activated or not. In the former case, we assumed that reference jackals and dogs were *a priori* correctly identified and assigned to their own clusters (popflag=1), while the putative hybrids were left to be assigned (popflag=0).

The software NewHybrids [42] was then used to compute the posterior probability for each genotype to belong to each of the six following classes: jackal (J) and dog (D) parentals, F_1_ and F_2_, backcrosses of F_1_ with dogs (BC1D) and with jackals (BC1J). Posterior distributions were evaluated after 10^5^ iterations of the Monte Carlo Markov chains, following a burn-in period of 10^4^ iterations, without any individual or allele frequency prior information, with ‘Jeffreys-like’ or ‘Uniform’ priors for mixing both proportions and allele frequencies.

In addition, we used Hybridlab [43] to evaluate the power of the 15 STRs to correctly detect *a priori* known parentals, hybrids and backcrosses. We used the 50 reference golden jackals and the 51 reference dogs to simulate 50 genotypes for each of the following classes: first and second generation hybrids (F_1_, F_2_), first and second generation backcrosses with golden jackal and dog (BC1J, BC1D, BC2J, BC2D). The simulated genotypes were analysed in Structure and NewHybrids using the run parameters described before (‘Admixture’ and the ‘I’ models, without any prior population information).

### 2.6 *K* locus analysis and major histocompatibility complex analyses

Since one putative hybrid showed a black coat (figure 3), we assessed the presence of the functional melanistic deletion at the *β*-defensin CBD103 gene (corresponding to the *K* locus), which determines black coat colours in dogs and wolves [16,27,44], following the amplification protocol described in Caniglia *et al*. [16] (electronic supplementary material, table S1).

The 50 golden jackal samples and the three putative hybrid samples were further analysed for MHC DLA-DRB1, DQA1 and DQB1 class II genes using cloning/sequencing method. The primers used to amplify exon 2 were: for DLA-DRB1, forward DRBF [45] and reverse DRB1R [46]; for DLA-DQA1, forward DQAin1 and reverse DQAIn2 [47]; for DLA-DQB1, forward DQB1BT7 [47] and reverse DQBR3, ACCTGGGTGGGGAGCCCG (primer designed in this study based on the sequence published in Wagner *et al*. [48]) (electronic supplementary material, table S1). Amplifications were carried out by PCR in a total volume of 25 μl containing 150–250 ng of genomic DNA, 1× QIAGEN HotStarTaq Master Mix (Qiagen, Hilden, Germany) (consisting of 1× PCR buffer, 200 μM of each dNTP and 2.5 units HotStartTaq DNA polymerase) and 0.2 μM of each primer. A negative control containing no DNA template was included in each amplification run to detect any contamination. All amplifications were performed using a standard touchdown PCR protocol as described in Kennedy *et al*. [49]. PCR products were visualized on 1% agarose gels stained with SYBR Safe DNA gel stain (Invitrogen) and purified by the Wizard SV Gel and PCR Purification Clean-Up System (Promega). Sequencing for typing was performed using the same primers as for PCR in reverse direction for all three loci. Confirmation of new alleles was performed by sequencing in both directions and by further DNA cloning. The PCR products were ligated into vectors and transformed into bacteria using the pGEM-T Vector System II (Promega). Plasmid DNA from 8 to 12 positive clones per individual was isolated using the Promega Wizard Plus SV Miniprep DNA Purification System, and inserts were sequenced using the PCR primers described above. Sequence processing and analysis were performed with BioEdit [30]. To identify alleles in heterozygous animals, we used the Applied Biosystems SeqScape® software, which is designed for analysis based on a locus-specific allele reference library, and previously described canid alleles that we obtained from the Immuno Polymorphism (IPD)—MHC Database (L. J. Kennedy 2013, personal communication), as described in Arbanasić *et al*. [50]. Three-locus haplotypes were unambiguously identified in a sequential process [51] and confirmed by Arlequin v. 3.11 [52].

## 3. Results

### 3.1 Mitochondrial DNA

We obtained fragments of 550 bp of the mtDNA CR from the three putative hybrid samples, which all carried sequences identical to the reference golden jackal mtDNA CR haplotype [18,19] (GenBank accession no. KF588364), suggesting their golden jackal maternal ancestry.

### 3.2 Y chromosome marker

Both male samples (S22 and 60c) produced two amplicons of the *Zfy* intron, characteristic of the domestic dog, suggesting their dog paternal ancestry.

### 3.3 Autosomal microsatellite loci

All the 15 STR loci were polymorphic in both reference populations, showing from three to 19 alleles in dog and from three to 10 alleles in jackal samples and an average number of alleles per locus of 8.2 (s.e. 0.98) and 4.3 (s.e. 0.50), respectively (electronic supplementary material, table S3). As expected, dogs showed an observed and expected heterozygosity higher than jackals and an average number of private alleles of 4.67 versus 1.07.

**Figure 4.**
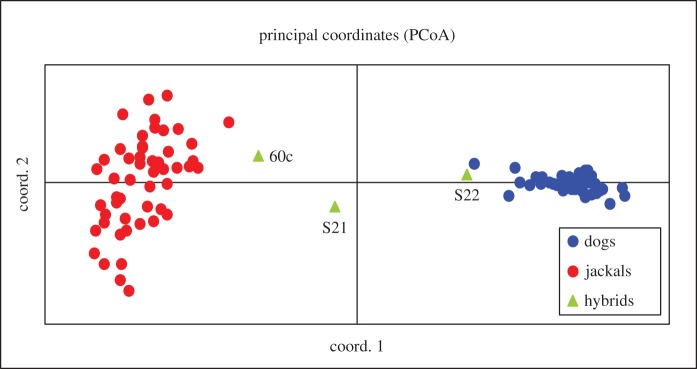
Principal coordinates analysis obtained by GenAlEx. The two principal axes (PC-I and PC-II) cumulatively explain 35.8% of the total genetic diversity. Blue dots represent the Croatian dog references, red dots the Croatian golden jackal references and triangles the golden jackal–dog hybrids: S21, S22 and 60c.

The PCoA results are shown in figure 4, where the individual scores were plotted onto the two principal axes (PC-I and PC-II), which cumulatively explain 35.8% of the total genetic diversity. The PCoA split jackals and dogs into two clearly separate clusters, with all dogs and jackals correctly identified by their genotypes. The only exception is three putative hybrids that showed intermediate positions between the two clusters, suggesting an admixed origin.

**Figure 5.**
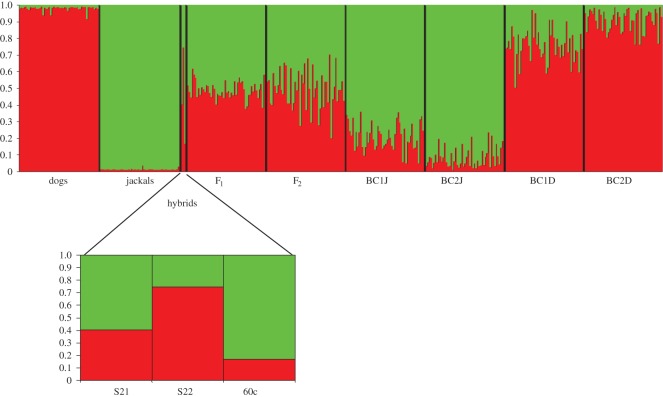
Bayesian analysis obtained by Structure using admixture models and *K*=2. Each individual is represented by a vertical bar fragmented in *K* sections of different length, according to their membership proportion in the two inferred genetic clusters: the red represent the dog component and green the jackal component. Dogs, Croatian dog references; jackals, Croatian golden jackal references; hybrids, golden jackal–dog hybrids; simulated genotypes by Hybridlab: F_1_ and F_2_, first and second generation hybrids, BC1J and BC2J, first and second backcrosses of F_1_ with golden jackals; BC1D and BC2D, first and second backcrosses of F_1_ with dogs.

At *K*=2 (the optimal number of genetic clusters), results from Structure admixture analyses showed that all dogs were assigned to a single cluster with an average membership proportion *Q*_D_ = 0.998 and individual proportions of admixture *q*_D_ ranging from 0.984 to 0.999. Jackals were assigned to the other cluster with *Q*_j_=0.999 and a *q*_J_ ranging from 0.994 to 0.999. The three putative hybrids S21, S22 and 60c showed *q*_J_=0.588 (90% confidence intervals CI: 0.396–0.770), 0.227 (0.063–0.411) and 0.849 (0.687–1.000), respectively (figure 5 and table 1).

**Table 1.** Assignment of observed and simulated dog, golden jackal and admixed genotypes to reference parental populations (dogs, jackals), their first (F_1_) and second (F_2_) generation hybrids, their first (BC1) and second (BC2) backcrosses with jackals and dogs. The assignments of individual genotypes of samples S21, S22 and 60C are also reported. *Q*_j_, average proportion of membership to jackal cluster; *q*_j_, proportion of membership to jackal cluster of individual genotypes; *P*, mean posterior probability for each population to belong to each of the six classes (D, dog; J jackal; F_1_, first generation hybrid; F_2_, second generation hybrid; BCD, backcrosses of F_1_ with dogs; BCJ, backcrosses of F_1_ with jackal); *p*, individual posterior probability to belong to each of the six classes. The highest values are marked in italic.

	Structure				NewHybrids					
	only observed		observed and simulated							
	*Q*_j_ (90% CI) Admixture model	*Q*_j_ (90% CI) UsePopInfo model	*Q*_j_ (90% CI) Admixture model	*Q*_j_ (90% CI) UsePopInfo model	*P*_D_	*P*_J_	*P*_F_1__	*P*_F_2__	*P*_BCD_	*P*_BCJ_
dogs	0.002 (0.000–0.013)	0.000	0.020 (0.000–0.087)	0.000	*0*.*991*	0.000	0.000	0.000	0.009	0.000
jackals	*0*.*999* (0.994–1.000)	*1*.*000*	*0*.*988* (0.943–1.000)	*1*.*000*	0.000	*0*.*998*	0.000	0.000	0.000	0.002
F_1_	—	—	0.511 (0.335–0.687)	0.508 (0.341–0.673)	0.000	0.000	*0*.*964*	0.006	0.004	0.026
F_2_	—	—	0.505 (0.331–0.678)	0.502 (0.338–0.666)	0.000	0.000	0.068	*0*.*666*	0.126	0.139
BC1J	—	—	0.789 (0.629–0.921)	0.754 (0.600–0.884)	0.000	0.061	0.011	0.013	0.000	*0*.*915*
BC2J	—	—	0.917 (0.796–0.988)	0.865 (0.737–0.960)	0.000	*0*.*560*	0.000	0.002	0.000	*0*.*438*
BC1D	—	—	0.243 (0.104–0.408)	0.269 (0.132–0.426)	0.058	0.000	0.077	0.025	*0*.*839*	0.000
BC2D	—	—	0.090 (0.014–0.215)	0.139 (0.041–0.270)	*0*.*547*	0.000	0.000	0.002	*0*.*451*	0.000

	*q*_j_ (90% CI) Admixture model	*q*_j_ (90% CI) UsePopInfo model	*q*_j_ (90% CI) Admixture model	*q*_j_ (90% CI) UsePopInfo model	*p*_D_	*p*_J_	*p*_F_1__	*p*_F_2__	*p*_BCD_	*p*_BCJ_
S21	0.588 (0.396–0.770)	0.561 (0.403–0.717)	0.598 (0.416–0.772)	0.586 (0.413–0.750)	0.000	0.000	*0*.*638*	0.075	0.002	0.285
S22	0.227 (0.063–0.411)	0.319 (0.166–0.477)	0.255 (0.105–0.429)	0.275 (0.130–0.440)	0.000	0.000	0.000	0.018	*0*.*982*	0.000
60c	0.849 (0.687–1.000)	0.735 (0.584–0.875)	0.832 (0.680–0.954)	0.791 (0.646–0.912)	0.000	0.005	0.000	0.003	0.000	*0*.*991*

When we assumed that reference jackals and dogs were *a priori* correctly identified and assigned to their own clusters (popflag=1), while the putative hybrids were left to be assigned (popflag=0), results obtained from five Structure runs using *K*=2, PopINFO and ‘I’ model were concordant with those obtained without any prior information (table 1).

Results of admixture analyses showed that 97.34% of the simulated admixed genotypes can be correctly identified as admixed at threshold *q*_i_=0.980 (after [53], we used as a threshold the minimum *q*_i_ value observed in reference populations using only real data: 0.984). All the F_1_, F_2_, BC1J and BC1D were correctly identified as admixed using both Structure models (Admixture and PopInfo). Only 6% of BC2J and 10% of BC2D genotypes showed a *q*_i_>0.980 (to jackal cluster and to dog cluster, respectively). Using the Admixture model with selection Flag to reference populations and PopInfo model, all simulated were correctly identified as admixed (figure 5).

In agreement with Structure results, the three putative hybrids were identified also by NewHybrids as admixed. S21 showed a posterior probability *p*=0.638 to belong to F_1_ class; S22 had *p*=0.982 to belong to backcross with dog and 60c had *p*=0.991 to be a backcross with jackal (table 1).

Relatedness analysis of female S21 and juvenile male S22 revealed that they shared at least one allele on each STR locus, confirming their mother–son relationship (electronic supplementary material, table S4).

### 3.4 *K* locus and major histocompatibility complex

The black hybrid 60c showed a heterozygote genotype at the *K* locus (*K*^+^/*K*^B^), whereas the deletion was absent in all reference jackals but present in 14 of the 51 reference dogs (table 2).

**Table 2.** Number and frequency (in parenthesis) of genotypes at the *β*-defensin CBD103 gene: *K*^+^/*K*^+^, homozygotes wild-type (no deletion); *K*^+^/*K*^B^, heterozygotes for the KB melanistic deletion; *K*^B^/*K*^B^, homozygotes for the KB melanistic deletion. *N*_a_, number of alleles; *N*_p_, number of private alleles; *H*_o_ and *H*_e_, observed and expected heterozygosity; HWE prob., probability test for Hardy–Weinberg equilibrium.

ref. pop (*N*)	*N*_a_ (s.e.)	*N*_p_ (s.e.)	*H*_o_ (s.e.)	*H*_e_ (s.e.)	HWE prob.	*K*^+^/*K*^+^	*K*^+^/*K*^B^	*K*^B^/*K*^B^
dogs (51)	8.20 (0.99)	4.67 (0.80)	0.61 (0.05)	0.68 (0.05)	0.0000	36 (0.72)	11 (0.22)	3 (0.06)
jackals (51)	4.33 (0.50)	1.07 (0.30)	0.46 (0.04)	0.54 (0.05)	0.0028	51 (1.0)	0	0

All 50 golden jackals and three putative golden jackal–dog hybrids successfully amplified at all three MHC loci analysed. In golden jackals, we found four DRB1, two DQA1 and two DQB1 alleles (table 3). Of these, three DLA-DRB1 (DLA-DRB1*13001, 13101 and 04503), one DLA-DQA1 (DLA-DQA1*03001) and both DLA-DQB1 alleles (DLA-DQB1*02305 and 06801) were not identified before in any canid species. New alleles were assigned official names by the DLA Nomenclature Committee (GenBank accession numbers KT159767, KT182924–KT182928). Identified alleles combined to form four DLA-DRB1/DQA1/DQB1 three-locus haplotypes (table 3). In all three putative golden jackal–dog hybrids, we identified the same DLA-DRB1*00901/DQA1*00402/DQB1*02305 haplotype, which was exclusive to and the most frequent among golden jackals in our reference samples. Among the three alleles that constitute this haplotype, the allele DQB1*02305 appears to be specific for golden jackals, whereas alleles DRB1*00901 and DQA1*00402 were previously found in dogs, but not in the same haplotype (L. J. Kennedy 2013, personal communication).

**Table 3.** DLA-DRB1/DQA1/DQB1 haplotypes found in 50 golden jackals and genotypes found in three golden jackal–dog hybrids. Alleles in bold were found exclusively in golden jackal. Italicized haplotype was predominant in golden jackal population.

		haplotypes identified in 50 golden jackal individuals			haplotype frequency (%)	no. of animals with the haplotype (no. of homozygotes)
golden jackal		DRB1	DQA1	DQB1		
		*00901*	*00402*	***02305***	50.00	35 (15)
		**13001**	00402	**02305**	30.00	24 (6)
		**13101**	**03001**	**06801**	15.00	13 (2)
		**04503**	00402	**02305**	5.00	4 (1)

	individual	genotypes identified in three golden jackal–dog hybrids			haplotype determination	
golden jackal–dog hybrids	S21	*00901*	*00402*	***02305***	golden jackal	
		00803	00301	00401	Croatian sheepdog, border terrier^a^	
	S22	*00901*	*00402*	***02305***	golden jackal	
		02001	00401	01303	more than 25 dog breeds [54]	
	60c	*00901*	*00402*	***02305***	golden jackal	
		00101	00101	00201	50 dog breeds [54]	

^a^L. J. Kennedy 2013, personal communication.

The other haplotype and alleles present in hybrids were not seen in this golden jackal cohort, but are common in dogs. The haplotype DLA-DRB1*00803/DQA1*00301/DQB1*00401 found in putative hybrid S21 was previously detected in the Croatian sheepdog and border terrier (L. J. Kennedy 2013, personal communication), while haplotypes DLA-DRB1*02001/DQA1*00401/DQB1*01301 and DLA-DRB1*00101/DQA1*00101/DQB1*00201 detected in putative hybrids S22 and 60c, respectively, were found in numerous dog breeds [54] (table 3).

## 4. Discussion

Using genetic markers we confirmed that the three individuals with anomalous phenotypic characters were indeed interspecific hybrids, namely a first generation hybrid between golden jackal and domestic dog (female S21), a backcross to dog (juvenile male S22) and a backcross to jackal (male 60c). The existence of backcrosses confirms that golden jackal–dog hybrids are fertile. Although these two species are estimated to have diverged about 1.7 Ma [2], or 0.4 Ma according to Freedman *et al*. [9], the occurrence of their hybrids and the fact that they are fertile do not come as a surprise, since reproductive isolation between pairs of geographically overlapping species evolves progressively [1] and may need hundreds to millions of generations to complete [55]. Golden jackals and dogs, together with grey wolves and coyotes, form a monophyletic clade and are more closely related than the golden jackal to two other jackal species, the side-striped jackal (*Canis adustus*) and the black-backed jackal (*Canis mesomelas*) [2]. Furthermore, Moura *et al*. [56] also reported weak evidence for current hybridization between grey wolves and golden jackals, as they identified several Bulgarian wolves exhibiting mixed ancestry with the jackal cluster.

The fact that golden jackal–dog hybrids were not recorded before in the wild might be a consequence of low research interest in golden jackals in the past, as they were not present in countries with the most active research communities. The golden jackal range has been expanding from southeastern Europe northwards and westwards in the last 30 years [8], thus a number of monitoring projects have been recently activated, increasing the possibility to record phenotypically abnormal individuals that might have always occurred in the populations. Moreover, before the advent of genetic tools, hybrids without a clear phenotypic signature could have gone undetected. On the other hand, the occurrence of golden jackal–dog hybrids might indeed be increasing because of several factors. First, higher population densities of golden jackals due to their recent expansion in the Balkan Peninsula [8] could enhance the encounters with stray and free-ranging dogs, which are quite abundant in Croatia and occur in the same areas where jackals live, hunt and gather food (I. Bošković 2012, unpublished data). It is known that the golden jackal is opportunistic in nature, primarily uses easily accessible human-derived food [57] and greatly benefits from the presence of agricultural surroundings [57,58]. Second, high mortality rates associated with jackal culling in Croatia [59], which mainly takes place from November to January and partially coincides with the jackal breeding season, can disrupt the social structure and promote hybridization with dogs, as suggested for wolf and coyote [5,60] or wolf and dog [16]. Yet, our results indicate that at least in Croatia introgressive hybridization between the two species is not a widespread phenomenon. We showed that Croatian golden jackals and dogs remain separated, forming two well-differentiated genetic entities where individuals are assigned to their respective cluster (figures 4 and 5), without significant ancestry in the other cluster.

Golden jackals exhibit lower genetic diversity measures than dogs (number of alleles, number of private alleles, observed and expected heterozygosity) (electronic supplementary material, table S3). This is in line with previous investigations (e.g. [6,61]), which compared diversity measures between grey wolves and dogs and consistently reported higher values in dogs, probably due to the multiple events of domestication and introgression during dog history [61], or to an ascertainment bias [62]. However, this pattern could be different when taking into account the genetic variability of single dog breeds, which show much less genetic variation than what can be observed across breeds or in village dogs [63–65].

In order to evaluate the power of the STR loci in the determination of golden jackal–dog hybrids, we also used simulated genotypes for six different hybrid classes. The analysis of the simulation results revealed that these 15 STRs are variable enough to detect 100% of parentals and F_1_, F_2_, BC1J and BC1D hybrids using a threshold of 0.98. Only 6% and 10% of second backcrosses with jackals and dogs, respectively, were undetected using the Admixture model and no other *a priori* information in the Bayesian assignment procedure. Thus, the 15 STRs seem to be reliable and powerful enough to detect hybrids between golden jackals and dogs.

The analysis of the mtDNA CR and of the *Zfy* intron on the Y chromosome appears to be diagnostic, since these markers proved to be fixed for different haplotypes in golden jackals and dogs (this study, [19,20]). All the three anomalous individuals carried a golden jackal mtDNA CR haplotype, whereas the two males showed Y chromosome marker amplicons characteristic of dogs [20], enabling us to deduce their lines of descent (electronic supplementary material, figure S1): a female golden jackal mated with a male domestic dog to produce the hybrid F_1_ female S21, which further mated with a domestic dog producing the backcross S22. The father of the male 60c was a golden jackal–dog hybrid, whose mother was a golden jackal and whose father was a dog, and he further mated with a female golden jackal. Accordingly, in both cases of F_1_ hybrids (one documented—S21, and the other deduced—the father of 60c, which was not sampled), hybridization took place between female golden jackals and male dogs, which is congruent with the sexual asymmetry present in most hybridizations between domestic dogs and wild *Canis* species, e.g. grey wolf [61] and Ethiopian wolf [3], though occasionally violated [15]. The finding of a heterozygote genotype at the *K* locus (*K*^+^/*K*^B^) in the backcross to that of a golden jackal 60c with black coat coloration, the absence of the melanistic *K* locus mutation in all reference jackals and its presence in 14 of the dog samples (table 2) suggest that this hybrid could have received the *K* locus deletion from dogs. In this way, golden jackal could join the panel of canid species that possibly derived their melanistic *K* locus mutation through hybridization with domestic dogs, namely grey wolves and coyotes [16,27]. Further, our findings cast doubts on the hypothesis of Ambarli & Bilgin [66], according to which melanism in the golden jackal they camera-trapped was due to an independent mutation instead of introgression from the domestic dog, and suggest that this individual might be another case of golden jackal–dog hybrid.

The MHC loci further confirmed that the three anomalous individuals described here were hybrids. Three out of four DLA-DRB1 alleles, one out of two DLA-DQA1 alleles and both DLA-DQB1 alleles found in golden jackals in this research (table 3) have not been identified before in any canid species. Thus, they could represent golden jackal private alleles that can be used as species-diagnostic markers. However, as the three hybrids in our research did not possess any of those jackal private alleles on DRB1 and DQA1 loci, the species (and hence, hybridization) determination could not have been performed using either of those two loci separately. Notwithstanding, the combined use of alleles on three loci enabled us to reliably confirm golden jackal ancestry in all three individuals in our research, since all of them possessed a three-locus haplotype that is specific and exclusive for golden jackals (DRB1*00901/DQA1*00402/DQB1*02305, table 3) and has not been found in dogs (L. J. Kennedy 2013, personal communication). In other words, even when alleles on particular MHC loci are not species-specific, their two- or three-locus haplotypes might prove to be, due to high levels of linkage disequilibrium across large stretches of this genomic region, where particular combinations of alleles at neighbouring loci are maintained by selection [67]. Further, the other haplotype possessed by each of the three individuals was characteristic of dogs and none of those was found in our reference jackal samples (table 3). In addition, none of the alleles comprising those haplotypes was found in jackals, indicating that even a single locus would suffice to confirm the dog ancestry in those samples. Likewise, for the confirmation of jackal ancestry in this research, the exclusive use of DQB1 locus would suffice since both alleles found in the reference jackal population are private. However, it would come as no surprise if additional alleles were found on that locus as more jackal samples become typed, and if some of them were to be shared between golden jackals and domestic dogs. This phenomenon, known as trans-species polymorphism, is characteristic of MHC genes, mainly occurs among closely related species and is a consequence of balancing selection, which acts on MHC genes over the long term and maintains ancestral polymorphism in descendant species [68]. Indeed, when trans-species polymorphism is present, analysing three-locus haplotypes instead of individual MHC loci should add power to detect possible introgression. The procedure for analysing MHC markers is quite straightforward, and time and cost efficient even when analysing three loci. Albeit they were applied as markers to detect hybridization in only two studies up to now [24,25], our results indicate that MHC genes can be used as suitable molecular markers for the identification of vertebrate species and for the determination of hybridization events, at least when data on reference parental populations are available and they are not closely related.

Hybridization might play an important role in the process of animal evolution, especially in rapidly changing environments. Hybridizing with dogs, golden jackals might increase their variability and thus facilitate their long-term adaptation. In addition, adaptive introgression may be facilitated for genes evolving under multi-allelic balancing selection, such as the vertebrate MHC system, where increased resistance to infectious diseases from adapted MHC variants might be transferred to closely related recipient species, as long as fertile hybrids can be formed [69,70]. This could be the case for the jackal, where standing variation to face new adaptive challenges, such as new parasites associated with human-related food sources, may be low but could be compensated by introgression from dogs, which have already adapted to human-related environments [70]. Adaptive introgression of MHC genes was recently suggested between two closely related species of newts [71] and from archaic to modern humans [72]. Sexual selection may also facilitate introgression of dog MHC alleles into the genomes of golden jackals, potentially contributing to resistance against currently prevalent parasites, as was proposed for three-spined sticklebacks (*Gasterosteus aculeatus*) [73]. It is thought that MHC-based mate choice may allow genes to cross species boundaries if parasite selection is strong enough that the benefits of hybridization overcome its costs [74].

Conversely, hybridization and introgression may also have harmful effects on the fitness of animal populations in the wild, causing loss of genetic diversity due to genetic homogenization and/or outbreeding depression in local populations [75]. In addition, possible cross-species transmission of canine diseases [76] might pose another risk for the expanding golden jackal populations.

In summary, in this paper we document the first occurrence of three cases of golden jackal–dog hybrids. However, the frequency of hybridization events, the extent of possible genetic introgression of dog genes into European golden jackal populations and the consequences on genetic diversity and population fitness (either beneficial or unfavourable) still remain to be investigated.

## Supplementary Material

Figure S1: Pedigrees of the three hybrids (shown in green). Circles represent females, squares represent males.

## Supplementary Material

Table S1. Primer sequences (forward and reverse), annealing temperatures (Ta) and product sizes (bp) for mtDNA, Y chromosome marker, K locus and DLA-DRB1, DQA1 and DQB1 genes

## Supplementary Material

Table S2. List of autosomal microsatellite loci used, with primer sequences (forward and reverse), annealing temperatures and product sizes (bp)

## Supplementary Material

Table S3. Genetic variability at 15 autosomal microsatellites in 51 domestic dogs and 50 golden jackals sampled in Croatia

## Supplementary Material

Table S4. STR genotypes of female S21 and juvenile male S22

## Data accessibility

The dataset supporting this article has been uploaded as part of the electronic supplementary material.
