# Supplementary material for: First evidence of hybridization between golden jackal (Canis aureus) and domestic dog (Canis familiaris) as revealed by genetic markers
Source: R Soc Open Sci. 2015 Dec 2;2(12):150450. doi: 10.1098/rsos.150450 (PMC4807452; doi:10.1098/rsos.150450)
Supplement: Figure S1: Pedigrees of the three hybrids (shown in green). Circles represent females, squares represent males. [file rsos150450supp1.pptx]

## Slide 1
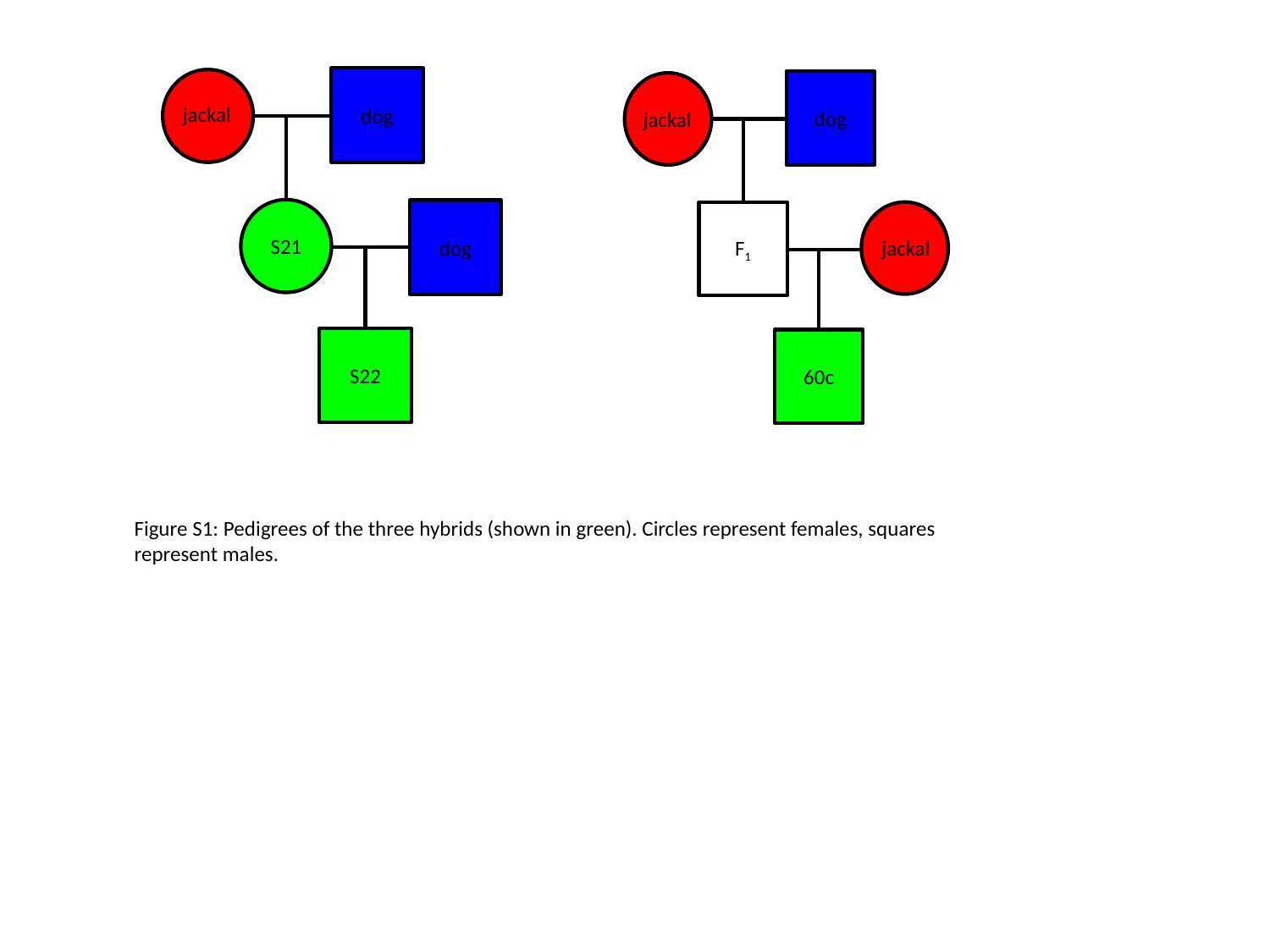

dog
S21
dog
S22
jackal
dog
F1
60c
jackal
jackal
Figure S1: Pedigrees of the three hybrids (shown in green). Circles represent females, squares represent males.
