# Supplementary material for: First evidence of hybridization between golden jackal (Canis aureus) and domestic dog (Canis familiaris) as revealed by genetic markers
Source: R Soc Open Sci. 2015 Dec 2;2(12):150450. doi: 10.1098/rsos.150450 (PMC4807452; doi:10.1098/rsos.150450)
Supplement: Table S1. Primer sequences (forward and reverse), annealing temperatures (Ta) and product sizes (bp) for mtDNA, Y chromosome marker, K locus and DLA-DRB1, DQA1 and DQB1 genes [file rsos150450supp2.docx]

Table S1. Primer sequences (forward and reverse), annealing temperatures (Ta) and product sizes (bp) for mtDNA, Y chromosome marker, K locus and DLA-DRB1, DQA1 and DQB1 genes

| **Marker** | **Product size** | **Ta** | **Forward / Reference** | **Reverse / Reference** |
| --- | --- | --- | --- | --- |
| mtDNA | 550 | 55°C | CGTCAGTCTCACCATCAACCCCCAAAGC  (L-Pro) / 1 | TTTGACTGCATTAGGGCCGCGACGG  (H576) / 2 |
|  |  |  |  |  |
| Y chromosome  marker | 536 (jackal)  242 + 566 (dog) | 57°C | GTCCATTGGATAATTCTTTCC  (Yint2-335) / 3  GCACTGCTAAATCAACCAC  (YintF2) / 4 | CAAGTTCTGCTTTGGTTCT  (YintR) / 4 |
|  |  |  |  |  |
| K-locus | 147-151 | 55°C | TGTCTTCATCCCTGTGAGGT / 5 | CCAGGAGGCATTTTCACACT / 5 |
|  |  |  |  |  |
| DLA-DRB1 | 267 | 62°C* | gatccccccgtccccacag  (DRBF) / 6 | tgtgtcacacacctcagcacca  (DRB1R) / 7 |
|  |  |  |  |  |
| DLA-DQA1 | 246 | 54°C* | taaggttcttttctccctct  (DQAin1) / 8 | ggacagattcagtgaagaga  DQAIn2 / 8 |
|  |  |  |  |  |
| DLA-DQB1 | 267 | 73°C* | ctcactggcccggctgtctc  (DQB1BT7) / 8 | ACCTGGGTGGGGAGCCCG  DQBR3 / 9 |
|  |  |  |  |  |

*Amplifications were performed using a touchdown PCR protocol consisting first of 95°C for 15 min, followed by 14 touchdown cycles comprising of 95°C for 30s, the annealing temperature for 1 min and 72°C for 1 min. Annealing temperatures were set initially at indicated temperatures, then reduced by 0.5°C in each cycle. This was followed by 20 cycles of 95°C for 30s; 55°C (DRB1), 47°C (DQA1) or 66°C (DQB1) for 1min and 72°C for 1 min. A final extension step was carried out at 72°C for 10 min.

[1] Douzery, E. & Randi, E. 1997 The mitochondrial control region of Cervidae: evolutionary patterns and phylogenetic content. *Mol. Biol. Evol.* **14**, 1154–1166.

[2] Randi, E., Lucchini, V., Christensen, M. F., Mucci, N., Funk, S. M., Dolf, G. & Loeschcke, V. 2000 Mitochondrial DNA variability in Italian and east European wolves: Detecting the consequences of small population size and hybridization. *Conserv. Biol.* **14**, 464–473.

[3] Shami, K. 2002 Evaluating the change in distribution of the eastern timber wolf (*Canis lycaon*) using the Y-chromosome. Master thesis, McMaster University. Cited in: Rutledge, L. Y., Garroway, C. J., Loveless, K. M., Patterson, B. R. 2010 Genetic differentiation of eastern wolves in Algonquin Park despite bridging gene flow between coyotes and grey wolves. *Heredity* **105**, 520-531.

[4] Galov, A., Sindičić, M., Gomerčić, T., Arbanasić, H., Baburić, M., Bošković, I. & Florijančić, T. 2014 PCR-based Y chromosome marker for discriminating between golden jackal (*Canis aureus*) and domestic dog (*Canis lupus familiaris*) paternal ancestry. *Conserv. Genet. Resour.* **6**, 275–277.

[5] Candille, S. I., Kaelin, C. B., Cattanach, B. M. et al. (2007) A *β*-defensin mutation causes black coat colour in domestic dogs. *Science* **318**:1418–1423.

[6] Kennedy, L. J., Quarmby, S., Fretwell, N., Martin, a. J., Jones, P. G., Jones, C. a. & Ollier, W. E. R. 2005 High-resolution characterization of the canine DLA-DRBI locus using reference strand-mediated conformational analysis. *J. Hered.* **96**, 836–842.

[7] Wagner, J. L., Burnett, R. C., Works, J. D. & Storb, R. 1996 Molecular analysis of DLA-DRBB1 polymorphism. *Tissue Antigens* **48**, 554–561.

[8] Wagner, J. L., Burnett, R. C., DeRose, S. A. & Storb, R. 1996 Molecular analysis and polymorphism of the DLA-DQA gene. *Tissue Antigens* **48**, 199–204.

[9] Primer designed in this study based on the sequence published in: Wagner, J. L., Hayes-Lattin, B., Works, J. D. & Storb, R. 1998 Molecular analysis and polymorphism of the DLA-DQB genes. *Tissue Antigens* **52**, 242–250.
