# Supplementary material for: First evidence of hybridization between golden jackal (Canis aureus) and domestic dog (Canis familiaris) as revealed by genetic markers
Source: R Soc Open Sci. 2015 Dec 2;2(12):150450. doi: 10.1098/rsos.150450 (PMC4807452; doi:10.1098/rsos.150450)
Supplement: Table S2. List of autosomal microsatellite loci used, with primer sequences (forward and reverse), annealing temperatures and product sizes (bp) [file rsos150450supp3.docx]

Table S2. List of autosomal microsatellite loci used, with primer sequences (forward and reverse), annealing temperatures and product sizes (bp)

| **Marker** | **Chr** | **Repeat** | **Allele Range** | **Temp.**  **Annealing** | **Multiplex** | **Forward** | **Reverse** | **Ref.** |
| --- | --- | --- | --- | --- | --- | --- | --- | --- |
| CPH6 | CFA23 | di | 120-126 | 57°C | A | CATTGGCTGTTTGACTCTAGG | ACTGATGTGGGTGTCTCTGC | 1 |
| FH2004 | CFA11 | tetra | 104-202 | 57°C | A | TCATTGCCTGATACAGACTGAG | TTGAGCTTAGTACTCAAAGCATAGTG | 2 |
| FH2088 | CFA15 | di | 91-139 | 57°C | A | CCCTCTGCCTACATCTCTGC | TAGGGCATGCATATAACCAGC | 2 |
| FH2140 | CFA5 | tetra | 112-168 | 57°C | B | GGGGAAGCCATTTTTAAAGC | TGACCCTCTGGCATCTAGGA | 3 |
| C20.253 | CFA20 | di | 90-120 | 57°C | B | AATGGCAGGATTTTCTTTTGC | ATCTTTGGACGAATGGATAAGG | 4 |
| CPH8 | CFA13 | di | 191-219 | 57°C | C | AGGCTCACAATCCCTCTCATA | TAGATTTGATACCTCCCTGAGTCC | 1 |
| FH2096 | CFA11 | tetra | 80-110 | 57°C | C | CCGTCTAAGAGCCTCCCAG | GACAAGGTTTCCTGGTTCCA | 2 |
| C09.250 | CFA9 | di | 117-145 | 57°C | D | TTAGTTAACCCAGCTCCCCCA | TCACCCTGTTAGCTGCTCAA | 4 |
| CXX.213 | CFA25 | di | 158-162 | 57°C | D | AATATGGGAGAGGAGAAGAGGG | ATGCTTCCTGGTAAGCAATCA | 4 |
| CPH4 | CFA15 | di | 130-155 | 57°C | E | ACTGGAGATGAAAACTGAAGATTATA | TTACAGGGGAAAGCCTCATT | 1 |
| CPH5 | CFA15 | di | 102-124 | 57°C | E | TCCATAACAAGACCCCAAAC | GGAGGTAGGGGTCAAAAGTT | 1 |
| CPH12 | CFA8 | di | 186-214 | 57°C | F | GGCATTACTTGGAGGGAGGAA | GATGATTCCTATGCTTCTTTGAG | 1 |
| CPH9 | CFA28 | di | 139-154 | 57°C | F | CAGAGACTGCCACTTTAAACACAC | AAAGTTCTCAAATACCATTGTGTTACA | 1 |
| FH2137 | CFA3 | di | 140-204 | 57°C | G | GCAGTCCCTTATTCCAACATG | CCCCAAGTTTTGCATCTGTT | 2 |
| CPH22 | CFA3 | di | 108-120 | 57°C | G | TCTTTCATTTACATTTTTGGCTCA | GCCCCAAAATCCGTGTGT | 5 |

[1] Fredholm M, Winterø AK (1995) Variation of short tandem repeats within and between species belonging to the Canidae family. Mammalian Genome 6:11-18.

[2] Francisco LV, Langston AA, Mellersh CS, Neal CL, Ostrander EA (1996) A class of highly polymorphic tetranucleotide repeats for canine genetic mapping. Mammalian Genome **7**:359–62.

[3] Breen M, Jouquand S, Renier C, et al (2001) Chromosome specific single-locus FISH probes allow anchorage of an 1800-marker integrated radiation-hybrid/linkage map of the domestic dog genome to all chromosomes. Genome Research 11:1784-1795.

[4] Ostrander EA, Sprague GF, Rine J (1993) Identification and Characterization of dinucleotide repeat (CA)*n* markers for genetic mapping in dog. Genomics 16:207-213.

[5] Dolf G, Schläpfer J, Gaillard C, Randi E, Lucchini V, Breitenmoser U, Stahlberger Saitbekova N (2000) Differentiation between Italian wolf and domestic dog based on microsatellite analysis. Genetics Selection Evolution 32:533–541.
