# Supplementary material for: First evidence of hybridization between golden jackal (Canis aureus) and domestic dog (Canis familiaris) as revealed by genetic markers
Source: R Soc Open Sci. 2015 Dec 2;2(12):150450. doi: 10.1098/rsos.150450 (PMC4807452; doi:10.1098/rsos.150450)
Supplement: Table S3. Genetic variability at 15 autosomal microsatellites in 51 domestic dogs and 50 golden jackals sampled in Croatia [file rsos150450supp4.docx]

Table S3. Genetic variability at 15 autosomal microsatellites in 51 domestic dogs and 50 golden jackals sampled in Croatia

| ***Dogs (51)*** | ***Locus ID*** | ***Na*** | ***Np*** | ***Ho*** | ***He*** | ***Prob*** | ***Signif*** |
| --- | --- | --- | --- | --- | --- | --- | --- |
|  | CPH4 | 6 | 1 | 0,56 | 0,70 | 0,098 | ns |
|  | CPH5 | 5 | 2 | 0,55 | 0,63 | 0,907 | ns |
|  | CPH6 | 11 | 7 | 0,79 | 0,85 | 0,000 | *** |
|  | CPH8 | 7 | 5 | 0,76 | 0,79 | 0,509 | ns |
|  | CPH9 | 8 | 4 | 0,65 | 0,64 | 0,863 | ns |
|  | CPH12 | 7 | 4 | 0,55 | 0,73 | 0,000 | *** |
|  | CPH22 | 3 | 1 | 0,13 | 0,21 | 0,005 | * |
|  | FH2004 | 11 | 6 | 0,75 | 0,78 | 0,027 | ns |
|  | FH2088 | 6 | 5 | 0,65 | 0,76 | 0,558 | ns |
|  | FH2096 | 4 | 0 | 0,50 | 0,52 | 0,947 | ns |
|  | FH2137 | 19 | 13 | 0,88 | 0,92 | 0,023 | ns |
|  | FH2140 | 9 | 5 | 0,77 | 0,77 | 0,812 | ns |
|  | CXX.213 | 9 | 6 | 0,49 | 0,79 | 0,001 | *** |
|  | C09.250 | 10 | 6 | 0,73 | 0,74 | 0,091 | ns |
|  | C20.253 | 8 | 5 | 0,37 | 0,41 | 0,388 | ns |
|  | **Average (SE)** | **8,20 (0,99)** | **4,67 (0,80)** | **0,61 (0,05)** | **0,68 (0,05)** |  |  |
|  |  |  |  |  |  |  |  |
| ***Jackals (50)*** | ***Locus ID*** | ***Na*** | ***Np*** | ***Ho*** | ***He*** | ***Prob*** | ***Signif*** |
|  | CPH4 | 4 | 1 | 0,63 | 0,66 | 0,743 | ns |
|  | CPH5 | 4 | 1 | 0,47 | 0,52 | 0,934 | ns |
|  | CPH6 | 3 | 0 | 0,45 | 0,53 | 0,350 | ns |
|  | CPH8 | 4 | 2 | 0,28 | 0,28 | 0,001 | ** |
|  | CPH9 | 4 | 0 | 0,51 | 0,53 | 0,525 | ns |
|  | CPH12 | 3 | 1 | 0,04 | 0,08 | 0,000 | *** |
|  | CPH22 | 3 | 1 | 0,31 | 0,46 | 0,063 | ns |
|  | FH2004 | 7 | 0 | 0,59 | 0,65 | 0,921 | ns |
|  | FH2088 | 2 | 0 | 0,43 | 0,49 | 0,389 | ns |
|  | FH2096 | 4 | 0 | 0,49 | 0,64 | 0,020 | ns |
|  | FH2137 | 10 | 4 | 0,63 | 0,76 | 0,002 | * |
|  | FH2140 | 4 | 1 | 0,57 | 0,71 | 0,222 | ns |
|  | CXX.213 | 3 | 0 | 0,35 | 0,39 | 0,326 | ns |
|  | C09.250 | 5 | 1 | 0,61 | 0,69 | 0,653 | ns |
|  | C20.253 | 5 | 3 | 0,57 | 0,71 | 0,134 | ns |
|  | **Average (SE)** | **4,33 (0,50)** | **1,07 (0,30)** | **0,46 (0,04)** | **0,54 (0,05)** |  |  |

Na = number of alleles; Np =number of private alleles; Ho and He = observed and expected heterozygosity; Prob = probability values of departures from Hardy-Weinberg proportions after Bonferroni correction: ns = not significant, * P < 0,05, ** P < 0,001, *** P < 0,0001.
