# Supplementary material for: First evidence of hybridization between golden jackal (Canis aureus) and domestic dog (Canis familiaris) as revealed by genetic markers
Source: R Soc Open Sci. 2015 Dec 2;2(12):150450. doi: 10.1098/rsos.150450 (PMC4807452; doi:10.1098/rsos.150450)
Supplement: Table S4. STR genotypes of female S21 and juvenile male S22 [file rsos150450supp5.docx]

Table S4. STR genotypes of female S21 and juvenile male S22

| locus | CPH4 | | CPH5 | | CPH6 | | CPH8 | | CPH9 | | CPH12 | | CPH22 | | FH2004 | |
| --- | --- | --- | --- | --- | --- | --- | --- | --- | --- | --- | --- | --- | --- | --- | --- | --- |
| S21 | 137 | **149** | 110 | **116** | **124** | **124** | **195** | **195** | 138 | **148** | 198 | **206** | 108 | **116** | 188 | 188 |
| S22 | 141 | **149** | 112 | **116** | **124** | **124** | **195** | **195** | 146 | **148** | 194 | **206** | 110 | **116** | 0 | 0 |

| locus | FH2088 | | FH2096 | | FH2137 | | FH2140 | | CXX.213 | | C09.250 | | C20.253 | |
| --- | --- | --- | --- | --- | --- | --- | --- | --- | --- | --- | --- | --- | --- | --- |
| S21 | **97** | **125** | 92 | **100** | **153** | 162 | 130 | **134** | **150** | 158 | 117 | **133** | 98 | **106** |
| S22 | **97** | **125** | 96 | **100** | **153** | 153 | 122 | **134** | **150** | 150 | 133 | **133** | 106 | **106** |

Shared genotypes are marked in bold.
